# An Incorrect Statement

**Published:** 1889-01

**Authors:** Ferdinand J. S. Gorgas

**Affiliations:** Dean.


AN INCORRECT STATEMENT.—In the printed proceedings of the Louisville meeting, August, 1888, of the "National Association of Dental Faculties," appears the following statement made before the said Association by Dr. B. Holly Smith, relating to the University of Maryland Dental Department:

"It is a hardship to be bound by our resolutions, when competing institutions near by are untrammeled. The College named (University of Maryland Dental Department) does not require a preliminary examination, while Colleges of this Association are compelled to do so."

If Dr. Smith had taken the trouble to examine the annual catalogues of the University of Maryland Dental Department for several years past, he would have found that a preliminary examination is required of every student before he is regularly matriculated, unless he can show by a diploma or other satisfactory evidence that he possesses a good English education. This preliminary examination as to proficiency in English branches has been exacted of every student, who could not present such evidence, for years past, and the statement of Dr. Smith was wholly incorrect. Every requirement published in our annual catalogues has been strictly enforced. Application for membership has been recently made to the "National Association of Dental Faculties" by the University of Maryland Dental Department, and we trust that hereafter no incorrect statements will appear concerning its curriculum, which has always been as high as that of any other school in this country.

FERDINAND J. S. GORGAS, Dean.

---

